# Refining Clinical Management Advice From Radiology for Intracerebral Haemorrhage: A Two-Cycle Clinical Audit With Intervening Service Improvement

**DOI:** 10.7759/cureus.117587

**Published:** 2026-10-07

**Authors:** Niha Mariam Hussain, David Swienton, David Eveson

**Affiliations:** 1 Radiology, University Hospitals of Leicester NHS Trust, Leicester, GBR; 2 Neuroradiology, University Hospitals of Leicester NHS Trust, Leicester, GBR; 3 Stroke Medicine, University Hospitals of Leicester NHS Trust, Leicester, GBR

**Keywords:** clinical audit, intracerebral haemorrhage, neurosurgical referral, radiology reporting, service improvement, stroke management

## Abstract

Spontaneous intracerebral haemorrhage (ICH) is a time-critical neurological emergency associated with high morbidity and mortality. Management guidelines recommend prompt blood pressure reduction, anticoagulation reversal where indicated, and early involvement of the stroke team, with no routine role for surgical intervention in small, deep, uncomplicated haemorrhages. At University Hospitals of Leicester NHS Trust, a pattern was identified in which radiology reports for this patient group frequently recommended neurosurgical consultation without associated guidance towards evidence-based medical management, contributing to delays in time-critical interventions. A two-cycle clinical audit with an intervening service improvement initiative was conducted to assess and improve the alignment of radiology reporting with guideline-directed management. Following a targeted educational intervention for reporting clinicians and promotion of a stroke-specific imaging request pathway, the distribution of management advice in radiology reports differed significantly between audit cycles, with inappropriate surgical referral advice eliminated entirely and appropriate medical management advice increasing from 17% to 52% of all reports. The rate of surgical consultation fell from 56% to 15%. Timely antihypertensive administration showed a non-significant improvement. These findings support targeting radiology reporting practice as a practical adjunct to broader ICH care bundle implementation.

## Introduction

Intracerebral haemorrhage (ICH) accounts for 15% of all strokes [1]. Recently, the third Intensive Care Bundle With Blood Pressure Reduction in Acute Cerebral Haemorrhage Trial (INTERACT3), principally conducted in low- and middle-income countries, demonstrated that a targeted care bundle for acute ICH with a principal focus on early physiological management, especially blood pressure reduction, was associated with improved functional outcomes at six months compared to standard of care [2]. Further trials are ongoing in high-income countries [3].

At University Hospitals of Leicester NHS Trust (UHL), where management guidelines for ICH reflecting the INTERACT3 protocol are available although there is no direct care bundle implementation, informal clinical observation by the audit team noted delays in the provision of these measures and frequent referral for surgical intervention. In particular, a common recommendation from radiology to seek surgical advice for ICH patients was noted without an associated recommendation for guideline-directed management.

Radiology reports are known to strongly influence clinical decision-making, particularly among resident doctors based in admissions units, who may rely on report conclusions to guide escalation and management [4,5]. Potential consequences include delayed blood pressure (BP) control, deferred stroke unit transfer, and unnecessary workload for neurosurgical teams who invariably recommend conservative management. Additionally, repeated inappropriate referrals risk contributing to a form of alarm fatigue, a well-recognised phenomenon in clinical settings whereby repeated low-yield alerts lead to diminished responsiveness over time [6]; applied to the referral context, it is plausible that a similar effect could reduce the perceived urgency of referrals that genuinely require neurosurgical input.

This two-cycle audit aimed to optimise acute ICH management by aligning radiology reporting practice with evidence-based guidelines. Specifically, our primary objective was to evaluate and improve the appropriateness of management advice within radiology reports, aiming to eliminate inappropriate neurosurgical referral recommendations through a targeted educational and pathway-based service intervention. Our secondary objective was to assess the downstream operational and clinical impacts of this reporting change, specifically regarding the reduction of unnecessary neurosurgical consultations and the timely administration of acute medical therapies (such as blood pressure-lowering medication) in the emergency setting.

## Materials and methods

We defined small, uncomplicated, deep intracerebral haemorrhage (sudICH) as a haematoma confined solely to deep cerebral structures (basal ganglia, thalamus, internal capsule, or hypothalamus), solitary, and without extension into the ventricular system, hydrocephalus, or involvement of lobar, cerebellar, or brainstem regions. In selecting this group, we could be certain that it was rational not to recommend surgical referral without an associated recommendation for guideline-directed management; both NICE Stroke Guidelines and the Royal College of Physicians’ Clinical Guidelines recommend that patients from this subgroup receive medical management prioritising tight BP control, with early stroke team involvement; surgical intervention is very rarely indicated in this group [7,8].

sudICH was identified retrospectively using Viz.ai (Viz.ai Inc., San Francisco, California), an AI-assisted CT triage tool that flags potential ICH on CT head imaging [9]. Cases flagged by the AI tool were manually reviewed against PACS imaging and clinical data to confirm eligibility. The consulting stroke physician on the team (DE) had direct clinical awareness of the relevant cases within the department during the study period and confirmed that all such cases had been captured by Viz.ai; no additional cases were identified through independent case-finding. Nonetheless, as cases were not independently searched through an alternative method, reliance on AI-assisted flagging remains a potential source of case-selection bias and is acknowledged as a limitation of the identified methodology. Data were collected retrospectively from electronic patient records (NerveCentre, Nervecentre Software Ltd., Wokingham, England).

The primary outcome was the proportion of radiology reports containing appropriate management advice, defined as a recommendation for medical management. Appropriate advice was defined as any report recommendation directing the clinical team towards medical management, including (but not limited to) stroke team involvement, blood pressure control, or conservative management. Inappropriate advice was defined as any recommendation for neurosurgical referral without accompanying guidance towards medical management. Absent advice was defined as reports containing no referral or management recommendation.

Secondary outcomes were the timeliness of antihypertensive administration, defined as the proportion of patients with an admission systolic BP of 140 mmHg or above (consistent with the threshold for treatment recommended by the NICE guideline NG128 [7]) receiving antihypertensives within one hour of hospital admission, and rates of surgical referral. In respect of management advice, reports were classified as appropriate, inappropriate, or absent.

Audit Cycle 1 (baseline, July-November 2024) was followed by a service improvement initiative comprising two categories of intervention. The first was educational: a Situation, Background, Assessment and Recommendation (SBAR) email communication endorsed by the Trust's head of neuroradiology, circulated to all reporting radiologists and radiographers, summarising the clinical evidence and management guidelines and providing explicit guidance on appropriate referral language. This was supplemented by teaching sessions attended by all reporting clinicians, reinforcing the evidence base for medical management and the limitations of surgical intervention in sudICH. Second, system-level changes were introduced: clinicians requesting CT head scans were encouraged to use a 'CT for Stroke' request pathway, which communicated stroke as the primary clinical concern to the reporting clinician and aimed to promote a stroke-first approach in report conclusions. Interventions were applied in December 2024, approximately six weeks prior to the commencement of Audit Cycle 2. Audit Cycle 2 (February-July 2025) was then conducted to assess the impact of these changes.

The project team comprised a foundation doctor working in acute admissions units (NH), a consultant stroke physician (DE), and a consultant neuroradiologist and lead of neuroradiology at UHL (DS). These roles represented some of the key stakeholders in the clinical pathway under evaluation: the reporting radiologist responsible for generating management advice, the stroke physician responsible for overseeing guideline-directed care, and the resident doctor working in acute admissions units who relies on radiology report conclusions to guide referral decisions. Reporting radiologists and radiographers across the department were engaged as participants in the educational interventions.

Statistical analyses were performed using Python (version 3.11; Python Software Foundation, Beaverton, Oregon) with the SciPy library (version 1.11). The distribution of management advice categories between cycles was assessed using a Monte Carlo simulated Fisher's exact test for the 3 × 2 contingency table, with 100,000 simulations performed using a fixed random seed of 42 to ensure reproducibility, and effect size quantified using Cramér's V. Pairwise comparisons for surgical referral rate and antihypertensive timeliness were assessed using a standard 2 × 2 Fisher's exact test. A p-value of <0.05 was considered statistically significant. Similarly, Wilson score confidence intervals were calculated for the principal proportions using Python with the SciPy library (version 1.11).

This project was formally registered through the University Hospitals of Leicester NHS Trust clinical audit governance process (registration number: 13712). As this project was not conducted as a service evaluation, formal ethics review was not required.

## Results

Audit Cycle 1 (July-November 2024, n = 18): The median patient age was 82 years (IQR 73-87 years); 12/18 (66.7%) were male. The proportion of reports containing appropriate management advice was 3/18 (16.7%, 95% CI 5.8-39.2%), with 10/18 (55.6%) containing inappropriate advice and 5/18 (28%) containing no advice. Surgical consultation was sought for 10/18 patients (55.6%, 95% CI 33.7-75.4%). In all referred cases, the neurosurgical team recommended conservative management, and no patient proceeded to surgery. Of 11 patients with an elevated admission BP, 6 (55%) received antihypertensive therapy within one hour of admission.

Audit Cycle 2 (February-July 2025, n = 27): The median patient age was 71 years (IQR 63-83 years); 13/27 (48.1%) were male. The proportion of reports containing appropriate management advice was 14/27 (51.9%, 95% CI 34.0-69.3%), with 0/27 (0%) containing inappropriate advice and 13/27 (48%) containing no advice. Of the reports containing no referral advice, 10/13 (76.9%) had been requested via the 'CT for Stroke' pathway, suggesting that the requesting clinician had identified stroke as a likely diagnosis. Surgical consultation was sought for 4/27 patients (14.8%, 95% CI 5.9-32.5%). As in Cycle 1, conservative management was recommended in all referred cases, and no patient proceeded to surgery. Of 22 patients with an elevated admission BP, 14 (64%) received antihypertensive therapy within one hour of admission.

The distribution of management advice categories differed significantly between cycles (Fisher's exact test, p < 0.0001; Cramér's V = 0.66, large effect size). The rate of surgical consultation fell from 55.6% to 14.8% (p = 0.007). Timely antihypertensive administration showed a non-significant improvement between cycles (54.5% to 63.6%, p = 0.714). Confidence intervals for the principal proportions are wide, reflecting the small sample sizes in both cycles, and should be interpreted with caution. Results are summarised in Table 1.

**Table 1:** Summary of reporting quality and clinical outcomes across both audit cycles

Outcome	Cycle 1 (n = 18)	Cycle 2 (n = 27)	P-Value
Radiology report			
Appropriate management advice	3/18 (17%)	14/27 (52%)	<0.0001
Inappropriate management advice	10/18 (56%)	0/27 (0%)	
No management advice	5/18 (28%)	13/27 (48%)	
Surgical consult sought	10/18 (55.6%)	4/27 (14.8%)	0.007
Appropriate antihypertensive administration within the first hour of admission	6/11 (54.5%)	14/22 (63.6%)	0.714

## Discussion

Provision of a care bundle for acute ICH whereby close attention is paid to rapid physiological management has been shown to improve patient outcomes in low- to middle-income countries. Whether this is an effective measure in high-income countries is unknown, but in our service (in a high-income setting without care bundle implementation), we found that, amongst patients with elevated admission BP, only around half were receiving timely antihypertensive treatment at baseline; this represents one component of guideline-directed ICH management rather than an overall measure of its delivery.

We studied a relevant adjunct to the broader stroke management bundle approach advocated by INTERACT3 [2]: the role of radiology reporting. Whilst INTERACT3 established that goal-directed care bundles improve functional outcomes in acute ICH, the effectiveness of such bundles depends on all members of the clinical team, including reporting radiologists, being aligned with management principles. We found that inappropriate surgical referral advice in radiology reports risked undermining bundle delivery by diverting clinical attention away from time-critical medical interventions. A simple educational intervention for this group, comprising an SBAR communication and targeted teaching sessions, was associated with closer alignment with guideline-directed management, with the distribution of management advice categories differing significantly between cycles (p < 0.0001, Cramér's V = 0.66). Prior work has demonstrated that standardisation of clinical pathways and education-based interventions can improve process metrics and adherence to guidelines in acute stroke care [3,10]; our findings are consistent with this evidence, and our educational intervention and promotion of a stroke-specific imaging request pathway represent pragmatic, low-resource examples of these approaches, extended here to the specific context of ICH radiology reporting.

The proportion of reports containing no management advice increased from 28% in the first audit cycle to 48% in the second cycle. Whilst this might initially appear to represent a limitation of the intervention, contextual analysis revealed that 10 of the 13 reports without advice in the second cycle had been requested via the ‘CT for Stroke’ pathway. This raises the possibility that the requesting clinician had already identified stroke as the primary diagnosis and that the absence of explicit referral guidance may have reflected shared clinical understanding between requester and reporter. However, this interpretation was not directly measured, and the absence of management advice may equally represent incomplete reporting. Both possibilities should be considered when interpreting this finding. Nevertheless, this finding highlights that the intervention did not consistently produce explicit appropriate advice across all reports, and a third audit cycle should assess whether routine inclusion of guideline-directed management advice can be embedded more reliably across all sudICH reports, regardless of request pathway.

The proportion of patients receiving antihypertensives within one hour did not statistically change between cycles (p = 0.714), and over a third of eligible patients did not receive timely BP-lowering medication post-intervention. It is likely that the study was underpowered to detect a statistically significant difference in this outcome given the small sample sizes, and the observed improvement from 55% to 64% may represent a clinically meaningful trend that a larger dataset would be better placed to evaluate. This reinforces the need for systems-level intervention rather than relying on education alone. The I-CATCHER trial, currently recruiting across UK NHS hospitals including UHL, will provide further evidence for the bundle approach in a UK context [3], and ensuring that radiology reporting practice supports rather than conflicts with bundle recommendations will likely be an important component.

Important limitations of this audit should be acknowledged. The sample sizes in both cycles were small, and the study was conducted at a single centre, limiting statistical precision and generalisability. Data on ICH severity (NIHSS scores), anticoagulation status, and discharge outcomes were not systematically collected (as they were rarely available during retrospective data collection) and would have provided a more complete characterisation of the patient population. Similarly, data on radiology report turnaround times were not available; understanding the interval between imaging acquisition, report issuance, and clinical decision-making would help clarify the practical influence of reporting recommendations on management behaviour. We aim to collect these data when completing future cycles of this audit.

As with all before-and-after clinical audits, the absence of a concurrent control group means that observed improvements cannot be definitively attributed to the interventions alone; secular changes in reporting practice or differences in case mix between periods may have contributed. The demographic differences between cycles, including the younger median age and higher proportion of female participants in Cycle 2, should be noted when interpreting the findings, though the primary outcome of this audit was reporting behaviour rather than patient-level outcomes. Additionally, as the educational and pathway-based interventions were implemented concurrently, it is not possible to determine the individual contribution of each component to the observed improvement in reporting quality. These are inherent features of the audit design, rather than unexpected methodological shortcomings, and are consistent with the scope and purpose of a service evaluation of this kind.

A further concern is sustainability: the improvements demonstrated here relied heavily on education delivered to a specific cohort of reporting clinicians, and high staff turnover in radiology (particularly at registrar level) means improvements may erode as new reporters rotate through without equivalent training. Embedding improvement into systems rather than individuals will therefore be critical. A third audit cycle is planned to assess the sustainability of these changes with a larger dataset. Further steps include expanding teaching to foundation doctors and acute care clinicians, formalising a care bundle for ICH, and re-auditing with a larger dataset to assess whether improvements are sustained over time.

## Conclusions

At UHL, radiology reporting practice was associated with clinical decision-making in ICH management. A two-cycle clinical audit with an intervening service improvement initiative demonstrated significant improvement in the alignment of reporting advice with evidence-based guidelines (p < 0.0001; Cramér's V = 0.66) and a clinically meaningful reduction in unnecessary surgical referrals (55.6% to 14.8%, p = 0.007). Timely antihypertensive administration showed a directional but non-significant improvement (54.5% to 63.6%, p = 0.714), highlighting the need for further systems-level intervention. These findings support targeting radiology reporting as a potential practical, low-resource adjunct to broader ICH care bundle implementation, and the approach warrants evaluation in other NHS centres.
